# A Quick Start Method for MEMS Disk Resonant Gyroscope

**DOI:** 10.3390/s21237986

**Published:** 2021-11-30

**Authors:** Xiaodong Xu, Xiaowei Liu, Yufeng Zhang

**Affiliations:** 1 MEMS Centre, Harbin Institute of Technology, Harbin 150000, China; bairixingchen@sina.cn (X.X.); xwl_hit_edu@126.com (X.L.); 2 Key Laboratory of Micro-Systems and Micro-Structures Manufacturing, Harbin Institute of Technology, Ministry of Education, Harbin 150000, China; 3 State Key Laboratory of Urban Water Resource & Environment, Harbin Institute of Technology, Harbin 150000, China

**Keywords:** disk resonator gyroscope, quick start, phase locked loop (PLL)

## Abstract

High-precision disk resonator gyroscope has a high quality factor in order to improve the performance of the gyroscope, as the high quality factor can lead to a long starting time. In this paper, a control system of the driving loop of the disk MEMS resonant gyroscope with the quick start is designed. The control system has functions of quick frequency locking and fast step response. Coarse-precision mode transition system is designed for quick frequency locking. A large-small mode transition system is designed for fast step response. The correctness of the design is verified by circuit test. The test results show that the start-up time is reduced by over 80% compared with the traditional control loop.

## 1. Introduction

Navigation includes astronomical navigation, satellite navigation, inertial navigation, radio navigation, etc., among which only inertial navigation is autonomous. Inertial navigation is difficult to disturb as it does not rely on external signals. Gyroscope and accelerometer are the core devices in the inertial navigation system. With the progress of micromachining technology, MEMS gyroscope has been widely used in fields like automotive, consumer electronics, industry, aerospace and other fields because of its small volume, low power consumption and low cost [1,2,3,4,5,6].

Disk resonant gyroscope (DRG) is a high-performance MEMS gyroscope which has attracted much attention in recent years [7,8,9]. It has the advantages of mode matching, high quantity factor, high impact resistance and being insensitive to environmental vibration [2,10]. With the increase of quality factor of the gyroscope, the start-up time also increases. Therefore, it is necessary to change the control system of driving mode to realize fast start of driving mode.

The start-up time of gyro driving mode mainly includes frequency locking time [11] and step response time. At present, most gyro driving mode control systems are using PLL (phase locked loop) to maintain stable operation [2,12,13,14]. In the digital phase-locked loop, the direct digital frequency synthesizer is used to generate sinusoidal signal to activate the gyro. There is deviation between the initial frequency of DDS (Direct digital frequency synthesizer) module and the resonant frequency of driving mode. Therefore, a phase-locked loop is needed to make the frequency of the output signal of DDS equal to the resonance of gyro driving mode. However, the amplitude of the output signal of the driving mode is very small at start-up, resulting in a long frequency locking time. In addition, the increase of the high quality factor of the gyro increases the step response time of driving mode [15].

Typical driving circuits use PLL to maintain the gyroscope vibrating at its resonant frequency. A quick frequency locking system with coarse-precision mode transition was proposed in paper [11]. The coarse resonance control loop is implemented using non-ideal differentiator, amplifiers and limiter for delaying the pickoff signal and a PLL is used for the precision loop. The control system is implemented by using analog electronics. A fully integrated excitation electronics for bulk micro machined gyroscopes was proposed in paper [16], which enables quick start-up or powerful continuous stage drive of a resonating sensor. The charge pump circuit converts the low voltage signal at the resonant frequency to a 20 V differential square wave to obtain large excitation force. Large excitation force can realize the quick start of gyroscope. A novel closed-loop drive circuit for the micro machined gyroscope has been presented in paper [17]. A comparator is used to instead of the AGC (Automatic gain control) circuit in this closed loop circuit to control the amplitude of the output signal, which overcomes the disadvantages of limited linear operational range and limited output signal amplitude. This method increases the excitation force and reduces the starting time. The method is simple and easily achieved. However, this control method cannot suppress the phase noise.

It can be seen that the method of quick start is mainly realized by increasing the excitation force. The control system is mainly implemented by using analog electronics. This paper introduces a method to realize the rapid start-up of dish vibratory gyroscope by adjusting the parameters of phase-locked loop in different stages. The control system realizes quick frequency locking and fast step response. A coarse precision mode conversion system is designed in this paper to shorten the frequency locking time. A large-small mode conversion system is designed to shorten the step response time of mechanical structure. This method enables the gyro to start faster. Compared with method in [11], the method in this paper uses less resources and is more suitable for digital circuit implementation.

In the second section, the working principle, frequency locking characteristics and step response characteristics of MEMS disk resonator gyro are introduced. In the third section, the design of quick frequency locking control system, fast response system and amplitude control system are introduced. In Section 4, the test results are introduced. The conclusion is presented in Section 5.

## 2. Architecture and Motion Model of Disk Gyroscope

### 2.1. Basic Structure of Mems Disk Resonator Gyroscope

**Figure 1:**
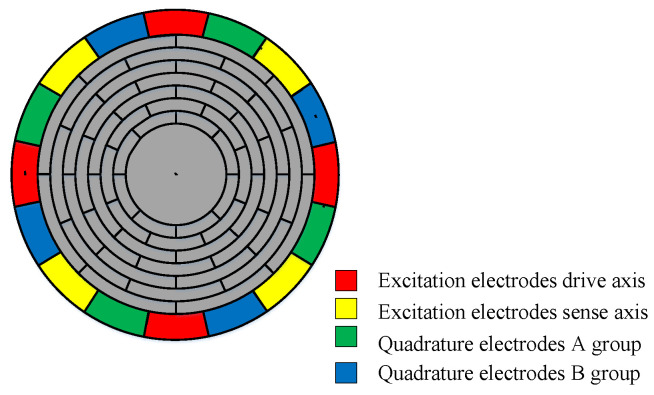
Structure of disk gyroscope.

The structure of MEMS disk resonant gyroscope is shown in Figure 1. The gyroscope is composed of concentric nested rings with spokes connected by spokes, and the resonator is supported by a central anchor. There are 16 electrodes around the disk resonator. There are also internal electrodes for electrostatic tuning [18,19,20].

The dynamic equations of driving mode and sensing mode are shown in Equation (1) [21,22,23]:

(1) $$\{\begin{array}{c} \ddot{x}+\frac{2}{\tau_{x}}\dot{x}+k_{12}y+\left(c_{12}-2A_{g}\Omega \right)\dot{y}+\omega_{x}^{2}x=f_{x}\\ \ddot{y}+\frac{2}{\tau_{y}}\dot{y}+k_{21}x+\left(c_{21}+2A_{g}\Omega \right)\dot{x}+\omega_{y}^{2}y=f_{y} \end{array}$$

where Ω is the input angular rate; *ω* and *k* are the resonant frequency and stiffness of each axis, respectively; *A_g_* is the angular gain; *τ* = 2*Q*/*ω*, *Q* is the quality factor.

The displacement of the driving mode can be written as:

(2) $$x=A\sin\omega_{d}t$$

**Figure 2:**
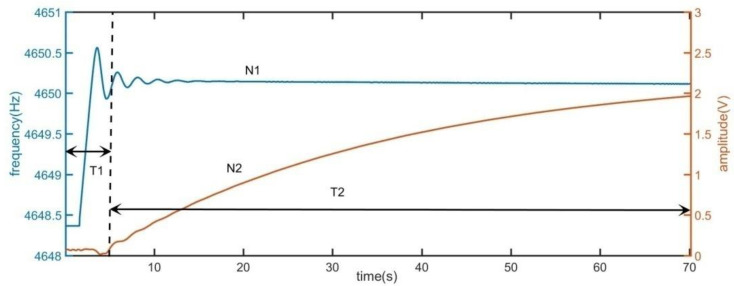
The start-up time of MEMS disk resonator gyroscope.

The start-up time of driving mode mainly includes two parts: frequency locking time and step response time. In the control system using PLL, the frequency locking time is the time when the frequency of the oscillator (DDS is generally used in digital control system) reaches the resonant frequency of the gyro driving mode from the starting frequency; when the frequency is locked, the gyro is in resonant state. The amplitude of the output signal of the driving mode begins to increase. This stage can be approximated as a step response. In Figure 2, the start-up time of MEMS disk resonator gyroscope is illustrated. The quality factor of gyro is more than 420,000.

As shown in the Figure 2, curve N1 represents the change of frequency and curve N2 represents the change of amplitude. T1 is the time of frequency locking and T2 is the time of step response. The difference between the initial frequency and resonant frequency of DDS module is 2 Hz. Therefore, at the initial time, the driving mode is in forced vibration. The amplitude of vibration is very low. As the output frequency of DDS module gradually approaches the resonant frequency, the driving mode is transiting to the resonant state. As shown in Figure 2, the output voltage of the driving mode gradually increases. It can be concluded that if the start-up time is required to reduce, the frequency locking time and the step response time of the driving mode are required to reduce respectively. The characteristics of frequency locking and step response of driving mode are analyzed below.

### 2.2. Analysis of Frequency Locking Characteristics

**Figure 3:**
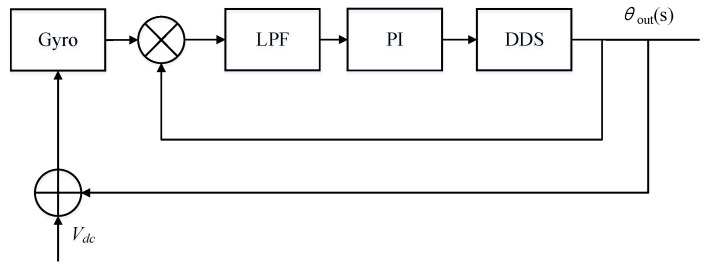
Principle of phase control loop.

Frequency locking is mainly realized by phase-locked control loop. The phase control loop is a servo system, which mainly realizes the locking and tracking control of frequency and phase. In order to analyze the characteristics of the phase control loop, the phase control loop of the gyro needs to be simplified. If the analog front stage, ADC (Analog to digital converter), DAC (Digital to analog converter) and amplitude control loop are ignored, a simplified phase control loop structure can be obtained, as shown in Figure 3.

**Figure 4:**
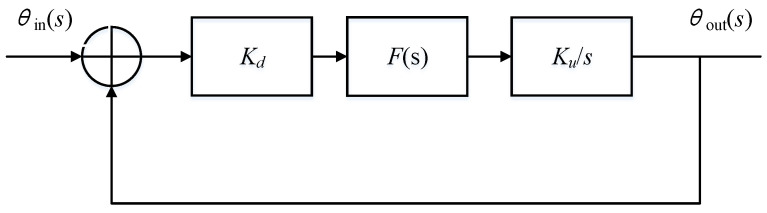
Principle of phase locked loop.

As shown in Figure 3 the phase difference between the driving mode output signal and the excitation signal is obtained by demodulation. The phase difference is input into the loop filter to obtain FTW (frequency tuning word). DDS is used to generate sinusoidal signal required for excitation. The structure is further equivalent to a control system as shown in Figure 4.

Where *K_d_* is the gain of the phase detector, *K_u_*/*s* is the frequency domain model of DDS, and *K_u_* is the gain of DDS. *K_d_* can be written as Equation (3)

(3) $$K_{d}=\frac{K_{d1}K_{d2}}{2}$$

where *K_d_*_1_ is the amplitude of the input signal and *K_d_*_2_ is the amplitude of the reference signal. The loop gain can be written as:

(4) $$G\left(s\right)=\frac{K_{d}K_{u}}{s}\frac{1+s\tau_{2}}{\tau_{1}s}$$

**Figure 5:**
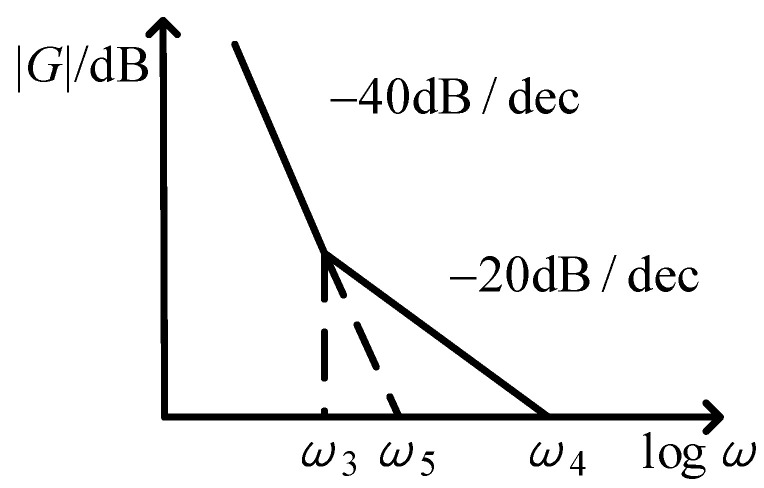
Frequency characteristics of phase control loop of driving mode.

The loop gain of phase control loop is related to the amplitude *K_d_*_1_ of the input signal and the gain *K_u_* of the DDS module. It can be represented as shown in Figure 5.

The DC gain of the loop is:

(5) $$\omega_{5}^{2}=\omega_{3}\omega_{4}=\frac{K_{d}K_{v}}{\tau_{1}}$$

The ratio of *ω*_4_ to *ω*_3_ determines the loop damping. When the ratio is 2, the damping is about 0.7. The bandwidth of PLL is:

(6) $$\omega_{4}=\sqrt{\frac{2K_{d}K_{v}}{\tau_{1}}}$$

By bringing Equation (3) into Equation (6), it can be derived:

(7) $$\omega_{4}=\sqrt{\frac{K_{d1}K_{d2}K_{v}}{\tau_{1}}}$$

The bandwidth of the loop is related to the amplitude of the input signal of the PLL. At startup, when the driving mode does not reach the resonant state, the amplitude of driving mode output signal *K_d_*_1_ is small. At this time, the frequency locking range is small. Due to the small loop gain, the frequency locking time is also long.

### 2.3. Analysis of Step Response Characteristics

**Figure 6:**
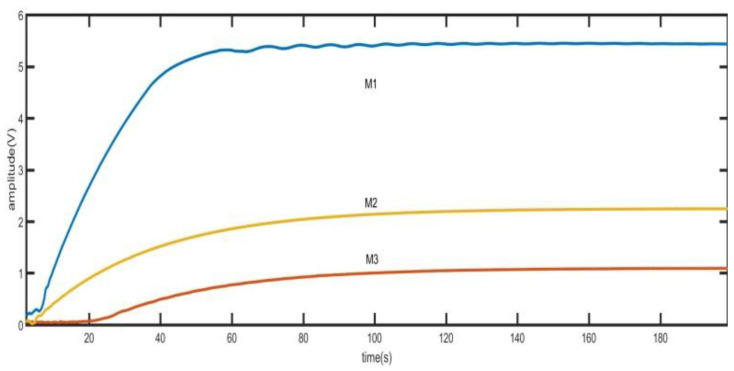
The time of step response.

When the frequency reaches or approaches the resonant frequency of the driving mode, the change of driving mode amplitude can be regarded as step response. It can be seen from Equation (1) that the driving mode can be approximated as a two-stage underdamped system. For an underdamped second-order system, the peak time, that is, the time required for the unit step response to reach the first peak, can be written as:

(8) $$t_{p}=\frac{\pi }{\omega_{d}}=\frac{\pi }{\omega_{n}\sqrt{1-\xi^{2}}}$$

where *ω_n_* is the undamped oscillation frequency and *ω_d_* is the damped oscillation frequency. The peak time is related to the gyro damping ratio. With the increasing quality factor of high-precision gyroscope, the start-up time of gyroscope will inevitably increase. As shown in Figure 6, the step response time of MEMS disk resonator gyroscope used in this paper is shown.

In Figure 6, M1, M2 and M3 are the voltage values of the output of the gyro driving mode under different driving forces. M1 has amplitude fluctuation near 5V due to output saturation caused by excessive driving force. And the amplitude is stabilized at 5V earlier because of output saturation. Under different driving forces, the time for the output to reach the stable value is approximately equal. The time in the three curves exceeds 80s, which is obviously unacceptable for the requirement of fast start. Therefore, the time of driving mode step response must be reduced.

## 3. Design of Control System for Quick Start of Driving Mode

The characteristics of frequency locking time and step response time can be obtained through the analysis in Section 2. This section introduces the design of a quick start control system.

### 3.1. Design of Quick Frequency Locking Control System

**Figure 7:**
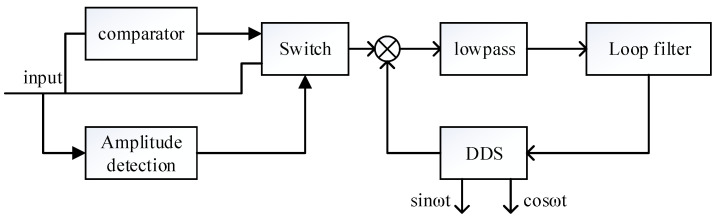
Phase control loop with quick frequency locking.

According to the analysis in Section 2.2, the reason for the long frequency locking time is that the output voltage of the driving mode is low at the initial stage of startup, which reduces the loop gain of the PLL. This will result in longer frequency locking time and cause the frequency locking range to become smaller or even unable to lock. Therefore, to solve this problem, the coarse-precision mode transition system shown in Figure 7 is introduced in this paper.

**Figure 8:**
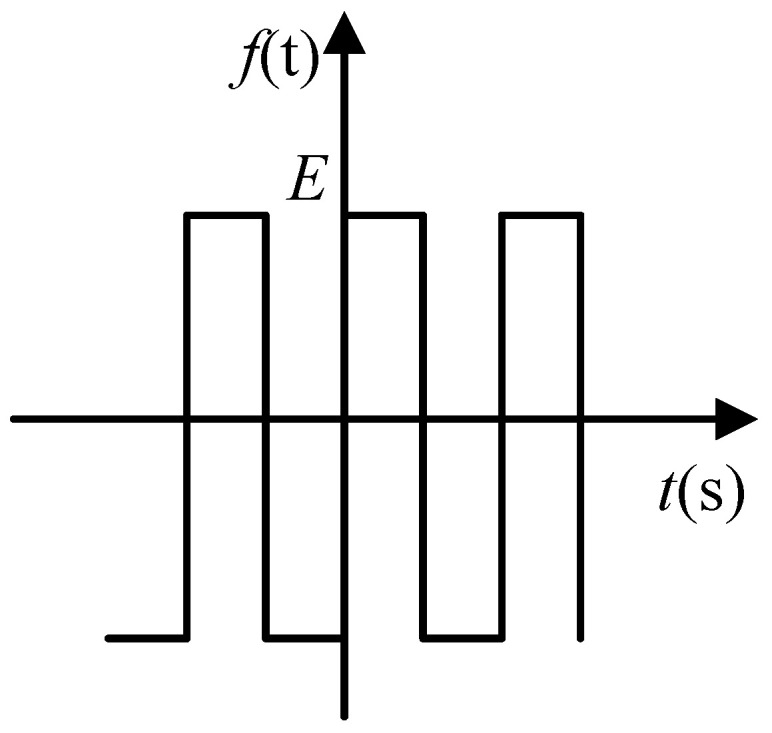
Square wave signal.

The difference from the traditional phase-locked loop is that the input of the phase detector in the phase-locked control loop is variable. In the initial stage of startup, the output signal of the gyro driving mode *x*_1_(*t*) is transformed into a square wave signal as shown in Figure 8. The square wave is used as the input of the phase detector to realize the quick locking of the phase control loop. After the frequency is approximately locked, the output signal of the gyro driving mode *x*_1_ (*t*) is used as the input of the phase detector to achieve more accurate locking and tracking.

The square wave signal *f* (*t*) converted by *x*_1_(*t*) is shown in Figure 8.

The square wave can be written as Equation (9)

(9) $$f\left(t\right)=\{\begin{array}{cc} E & x_{1}\left(t\right)\geq 0\\ -E & x_{1}\left(t\right)<0 \end{array}$$

where *E* is the amplitude of the square wave signal, and it can be obtained by Fourier expansion of the square wave signal.

(10) $$f\left(t\right)=\frac{4E}{\pi }\left[\sin2\pi ft+\frac{1}{3}\sin6\pi ft+\frac{1}{5}\sin10\pi ft+\cdots +\frac{1}{n}\sin2n\pi ft+\cdots \right]$$

Since the subsequent digital signal processing contains a digital filter, the high-frequency term can be filtered after demodulation, so *f* (*t*) can be approximately written as:

(11) $$f\left(t\right)\approx \frac{4E}{\pi }\sin2\pi ft$$

The demodulation is realized by multiplier. In this design, due to the use of sigma delta ADC, there is a down sampling process. The ADC data output rate is much lower than the sampling rate, so the multiplier can be realized by serial structure, which is simple in structure and occupies less resource. The demodulation result is shown in Equation (12).

(12) $$\begin{array}{l} f_{d}\left(t\right)=\frac{4E}{\pi }\sin2\pi ft\times K_{d2}\cos\left(2\pi f+\varphi_{0}\right)\\ =\frac{2E}{\pi }K_{d2}\sin\left(4\pi ft+\varphi_{0}\right)+\frac{2E}{\pi }K_{d2}\sin\left(\varphi_{0}\right) \end{array}$$

Since the subsequent digital signal processing contains a digital filter, the high-frequency term can be filtered after demodulation. The gain of the phase discriminator can be written as

(13) $$K_{d2}\approx \frac{2EK_{d2}}{\pi }$$

At this time, the loop gain of the PLL can be written as:

(14) $$G\left(s\right)=\frac{2EK_{d2}K_{v}}{\pi s}\frac{1+s\tau_{2}}{\tau_{1}s}$$

The bandwidth of PLL is:

(15) $$\omega_{4}=\sqrt{\frac{2EK_{d2}K_{v}}{\pi \tau_{1}}}$$

The loop gain of PLL is no longer affected by the amplitude of input signal. The locking time of the loop can be changed by changing the value of *E*. When the frequency is approximately locked, the loop is converted to accurate locking. At this time, the input of the PLL is no longer the converted square wave signal, but the signal before conversion. The amplitude of the amplitude control loop is used as the switching signal. It will be converted when the amplitude of the driving mode is greater than the set value. The amplitude control loop is introduced in Section 3.3.

### 3.2. Design of Quick Step Response Control System

**Figure 9:**
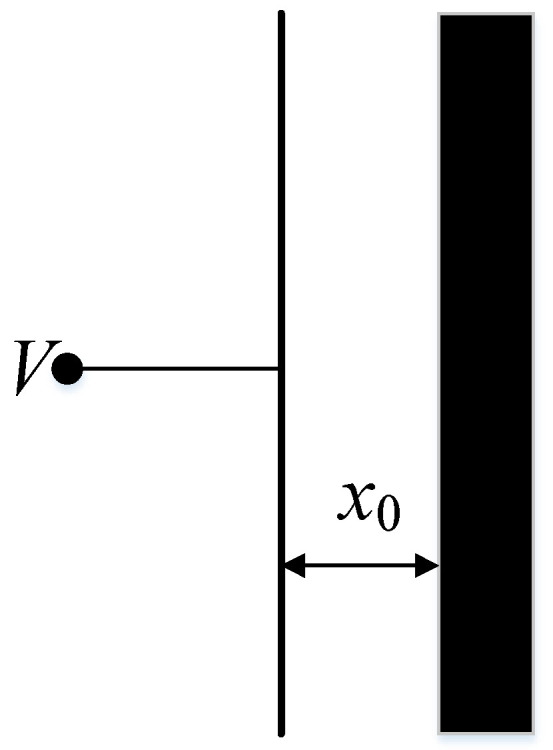
Model of excitation capacitance.

The MEMS disk resonator gyroscope uses capacitive excitation, and its equivalent structure is shown in Figure 9. The capacitance formed by the resonator and the excitation electrode can be regarded as a flat plate capacitance. The capacitance can be written as:

(16) $$C_{1}=\frac{A\varepsilon_{0}\varepsilon_{r}}{x_{0}+x}$$

where *x_0_* is the initial plate spacing and *x* is the moving distance. As shown in Figure 1 and Figure 9, for the vibration mode *n* = 2, capacitors with an angle difference of 90° have opposite displacements to each other. The capacitance can be written as:

(17) $$C_{2}=\frac{A\varepsilon_{0}\varepsilon_{r}}{x_{0}-x}$$

In signal processing, the difference between the two changes can be obtained to increase the signal amplitude. When x << x_0_, the variation of capacitance can be obtained as Equation (18).

(18) $$\Delta C=C_{1}-C_{2}=\frac{A\varepsilon_{0}\varepsilon_{r}}{x_{0}-x}-\frac{A\varepsilon_{0}\varepsilon_{r}}{x_{0}+x}\approx \frac{2A\varepsilon_{0}\varepsilon_{r}}{x_{0}^{2}}x$$

A voltage of *V*_1_ is applied to the excitation electrode and a voltage of *V_p_* is applied to the mass block, so that the voltage difference between the electrode and the mass block is:

(19) $$V=V_{P}-V_{1}$$

Therefore, the energy stored in the capacitor is:

(20) $$E=\frac{1}{2}CV^{2}=\frac{A\varepsilon_{0}\varepsilon_{r}V^{2}}{2\left(x_{0}+x\right)}$$

Electrostatic force can be written as:

(21) $$F=-\frac{\partial E}{\partial x}=-\frac{A\varepsilon_{0}\varepsilon_{r}V^{2}}{2{\left(x_{0}+x\right)}^{2}}$$

The differential excitation voltages *V_dc_* + *V_ac_*sin*ω_d_t* and *V_dc_* − *V_ac_*sin*ω_d_t* are applied to the electrodes respectively, and the resulting driving force is:

(22) $$\begin{array}{l} F_{tot}=\frac{A\varepsilon_{0}\varepsilon_{r}}{2{\left(x_{0}-x\right)}^{2}}{\left(V_{dc}+V_{ac}\sin\omega_{d}t\right)}^{2}-\frac{A\varepsilon_{0}\varepsilon_{r}}{2{\left(x_{0}+x\right)}^{2}}{\left(V_{dc}-V_{ac}\sin\omega_{d}t\right)}^{2}\\ \approx \frac{2A\varepsilon_{0}\varepsilon_{r}V_{dc}V_{ac}}{x_{0}{}^{2}}\sin\omega_{d}t \end{array}$$

According to Equation (8), the step response time of driving mode is fixed under fixed driving force. Therefore, the time of step response can be reduced by converting the driving force. Equation (23) gives the expression of the driving force, which can be simplified to obtain:

(23) $$F_{tot}\propto V_{dc}V_{ac}\sin\omega_{d}t$$

**Figure 10:**
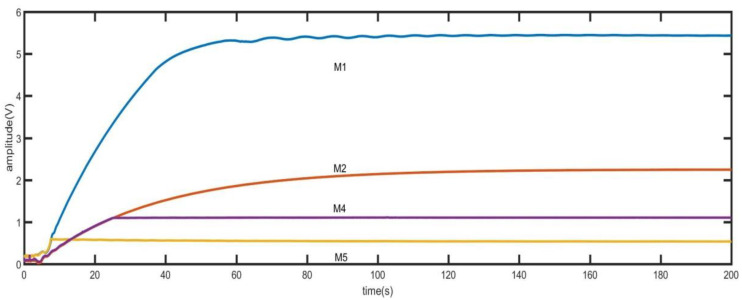
The time of quick step response.

The driving force is proportional to DC voltage *V_dc_* and AC amplitude *V_ac_*. *V_dc_* is mainly used for amplitude adjustment. The increase or decrease of the value is limited. As the digital control circuit is used in this paper, sinusoidal digital signal is mainly generated by DDS module, and then it converts to corresponding analog signal through DAC. Therefore, it can easily realize the multiplication change of *V_ac_* by shift operation. As shown in Figure 10, quick step response is realized by changing the value of *V_ac_*.

As shown in Figure 10, M1, M2 and M3 are the original step response curves, and M4 and M5 are the output curves corresponding to the quick step response. Taking M5 as an example, in the initial stage, the *V_ac_* in M5 is the same as that in M1, so it has the same upward slope in the initial stage. After reaching the predetermined value, the value of *V_ac_* is reduced to make it stable quickly.

It should be noted that whether it is quick frequency locking or quick step response, the control loop is always a servo system, so the stability of the system must be considered. In Section 3.1, the loop gain of the quick frequency locking loop is conducted, which is simplified as follows:

(24) $$G\left(s\right)\propto 2EK_{d2}$$

**Figure 11:**
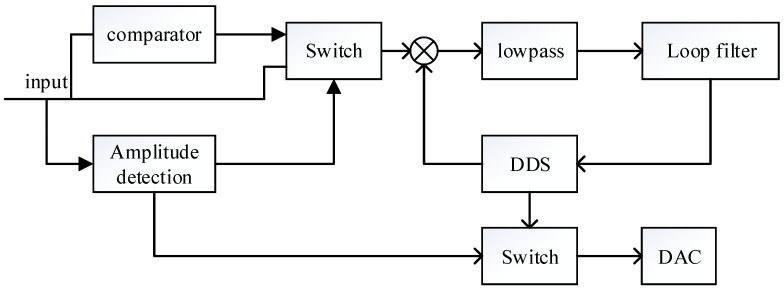
Control system of quick start driving loop.

The loop gain is proportional to *E* and *K*_d2_. In the design of quick step response, *V_ac_* is increased at the initial stage of start-up, which will inevitably lead to the increase of *K_d_*_2_ in the same proportion, resulting in the increase of loop gain *G*(*s*). There will be a certain margin in the design of phase loop. The increase of *V_ac_* leads to insufficient margin. This will result in the instability of phase control loop. Therefore, at the initial stage of step response, the relative invariance of *G*(*s*) must be guaranteed while increasing *V_ac_*. Therefore, it is necessary to reduce the value of *E* at this stage. After switching to a smaller *V_ac_*, *E* can be restored to its original size. Therefore, the quick locking circuit in Section 3.1 is improved and the structure diagram is shown in Figure 11. Figure 11 is the final version of the quick start method in this paper. It can realize fast frequency locking and fast step response. Compared with Section 3.1, the control of the amplitude of the signal input to the DAC is increased, and the *E* value is changed synchronously. The test results of this circuit will be given in Section 4.

### 3.3. Control System of Amplitude Stable Loop

**Figure 12:**
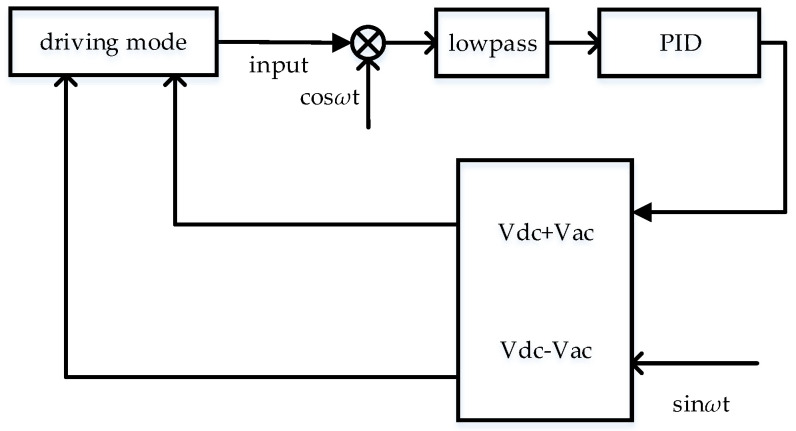
Amplitude control loop.

Gyro control system needs amplitude control loop to avoid change of amplitude caused by noise. In addition, the quick frequency locking circuit and fast step response circuit need to judge whether to convert the mode by monitoring the amplitude of the driving mode signal. The structure of amplitude control circuit is shown in Figure 12. The excitation voltages *V_dc_* +*V_ac_*sin*ωt* and *V_dc_* − *V_ac_*sin*ωt* are applied to the electrodes of the gyro driving mode. The amplitude of gyro output signal is controlled by PI controller.

## 4. Circuit Realization and Test

**Figure 13:**
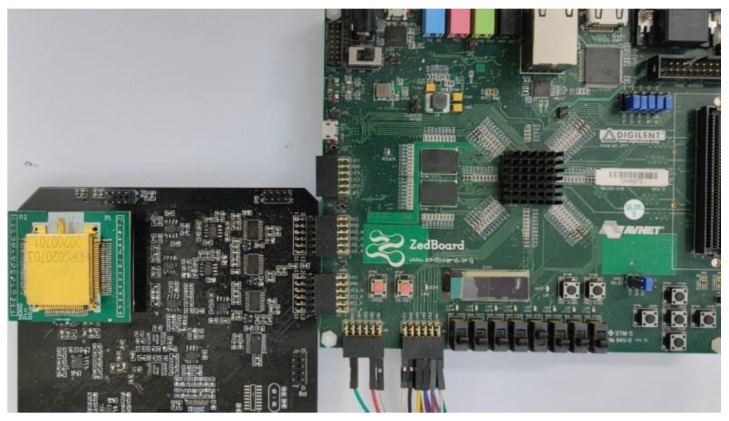
Gyro control circuit.

According to the above analysis, the whole gyro system is implemented at PCB level. The assembled PCB is shown in Figure 13. The charge amplifier, ADC, DAC and other modules are built by discrete devices, and the digital signal processing is implemented by FPGA.

**Figure 14:**
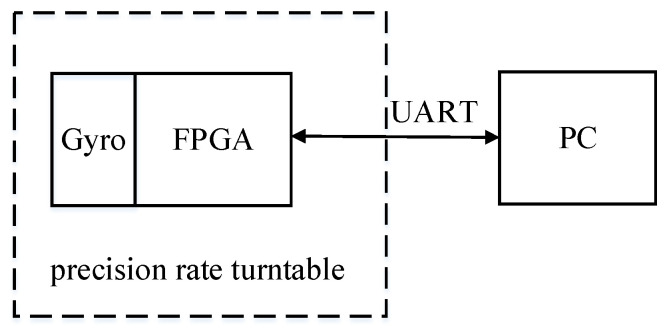
Test platform.

The test platform is shown in Figure 14. The value of the register in FPGA can be output to PC through serial port. The frequency tuning word of DDS module, that is, the vibration frequency of driving mode, can be output in this way. The amplitude detected by the amplitude control loop can also be output in this way.

Firstly, the quick locking control system is tested, and the frequency tuning word of DDS is output to PC. Convert the output value to the corresponding frequency.

**Figure 15:**
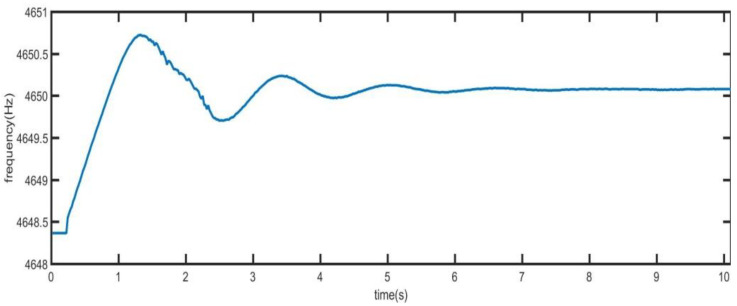
Vibration frequency of driving mode.

In Figure 15, the difference between the initial frequency and the resonant frequency of the driving mode is 2 Hz. At the beginning, the PLL is in the coarse locking state, and the frequency can be locked quickly. At this time, the frequency has large jitter. When the amplitude of the driving mode increases to the set value, the loop turns to precise locking. At this time, the accuracy of the phase-locked loop becomes higher.

**Figure 16:**
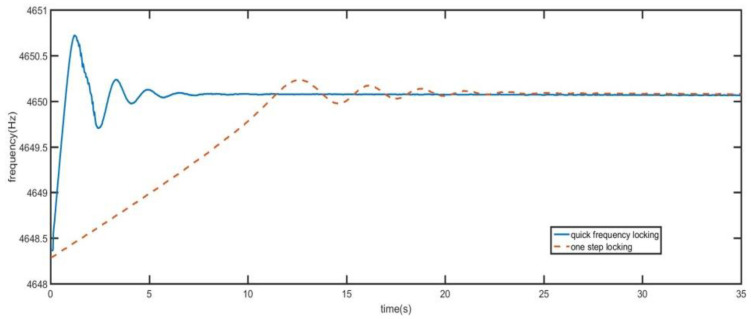
Comparison of two control modes when the initial frequency deviation is 1Hz.

**Figure 17:**
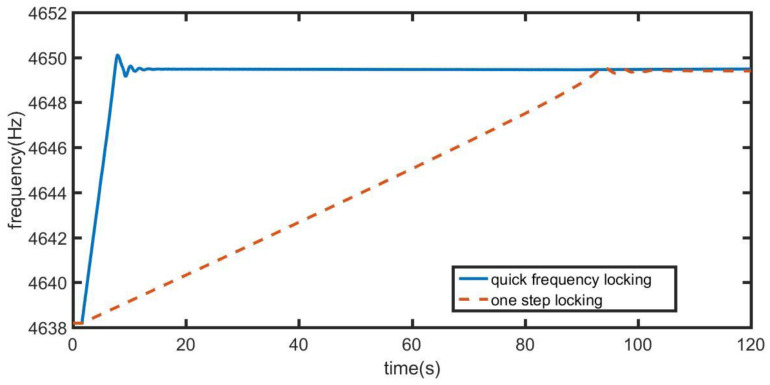
Comparison of two control modes when the initial frequency deviation is 10Hz.

As shown in Figure 16 and Figure 17, the phase-locked loop using quick frequency locking control system is compared with the loop using only precise locking control system. In Figure 16, when the initial frequency difference is 2 Hz, the frequency locking time of quick frequency locking control system is less than 8 s and the time of one-step locking control system is 25 s. Using quick frequency locking control system can achieve frequency locking in a faster time. Compared with the single step locking mode, the locking time using quick frequency locking control system is reduced by 68%. Moreover, after conversion to precise locking, the two modes have the same frequency accuracy.

From Figure 17, when the initial frequency difference is 10 Hz, the frequency locking time of quick frequency locking control system is 15 s. The frequency locking time of one-step locking is 90 s.

**Figure 18:**
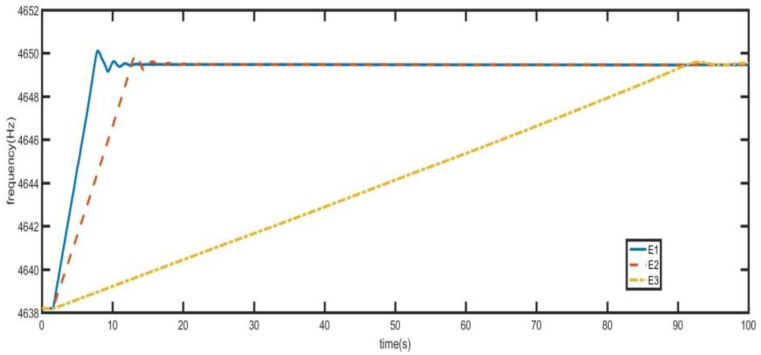
Vibration frequencies of driving modes under different parameters.

From the previous analysis, the time required for the coarse resonance control mode can be changed by changing the value of E in Equation (14). As shown in Figure 18, the initial frequency difference is 10 Hz. When *E* takes different values, the time required for quick frequency locking control system is different. In Figure 18, *E*1:*E*2:*E*3 = 16:8:1. With the increase of *E*, the initial frequency jitter is greater and the loop locking time is shorter.

**Figure 19:**
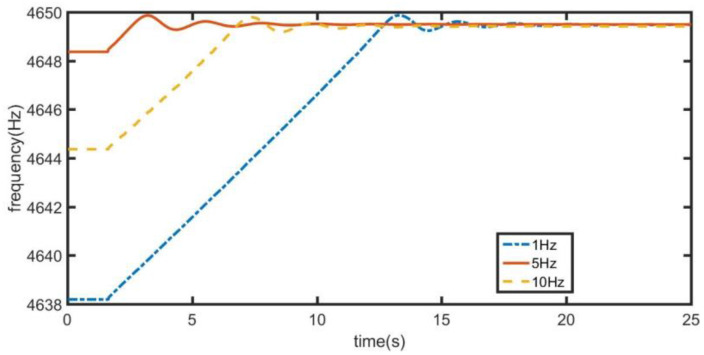
Variation of vibration frequency of driving mode under different initial frequencies.

From Figure 19, the change of vibration frequency of driving mode under different initial frequencies can be obtained. When *E* does not change and the initial frequency offset is 1 Hz, 5 Hz and 10 Hz, respectively, the slope of frequency change in the three startup stages is the same, and the startup time increases with the increase of initial frequency offset.

**Figure 20:**
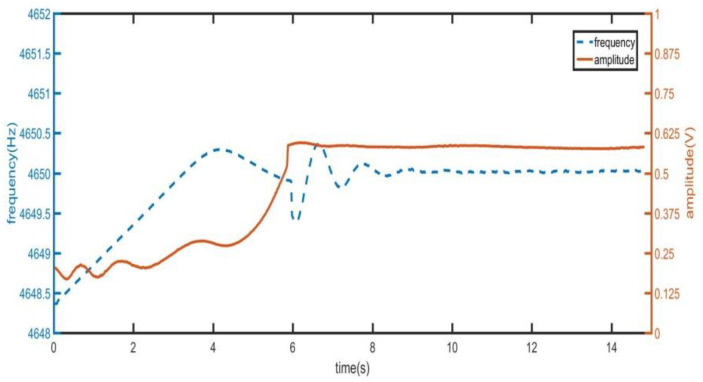
Changes in frequency and amplitude at start-up.

The test of fast step response has been given in the Figure 10. The Figure 20 shows the change of the amplitude of the output signal of the driving mode when the initial frequency deviation is 2 Hz.

**Figure 21:**
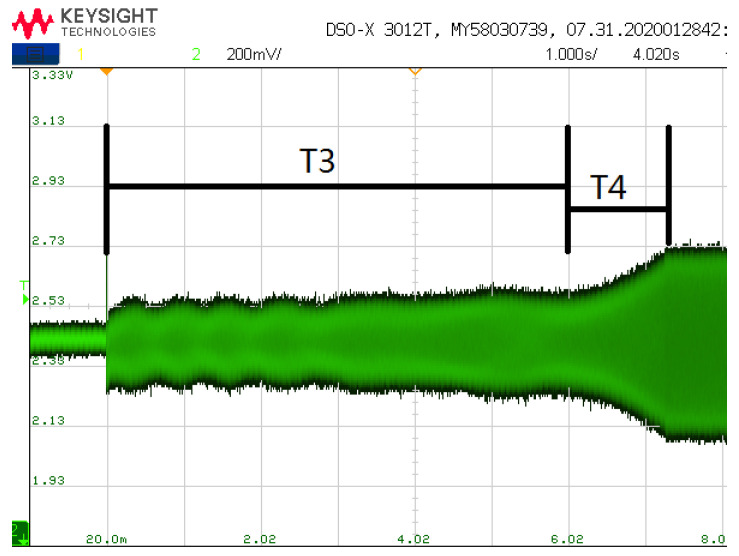
Amplitude change during start-up.

As shown in Figure 20, the frequency and amplitude variation diagram of driving mode tested by quick frequency locking and fast step response method when the initial frequency deviation is 2 Hz. It can be seen from the Figure 20 that when the frequency difference is 2 Hz, the amplitude of the output signal of the driving mode is very small. When the frequency is approximately locked, the amplitude of the signal begins to increase rapidly. After increasing to the set value, the output signal is quickly stabilized at a small amplitude value by switching vac. The switching of *V_ac_* also brings frequency jitter. At 9 s, the frequency and phase stability have been realized. Figure 6 has shown the variation of amplitude when using the traditional method. It can be seen that when using the traditional method, the start-up time is greater than 80 s. Comparing Figure 6 and Figure 20, the fast start-up method in this paper reduces the start-up time by more than 80%. The time domain waveform observed by the oscilloscope is shown in Figure 21, where T3 is the time of frequency locking and T4 is the time of step response.

## 5. Conclusions

A quick frequency locking circuit and a fast step response circuit are proposed in this paper in order to realize quick start of gyro driving mode. When the frequency is not locked, the coarse-precision mode transition system is used to realize quick frequency locking. In the initial stage of frequency locking, the coarse locking method is adopted to increase the frequency locking range of the gyro and reduce the frequency locking time. After the frequency becomes stable, the control mode is switched to precise frequency locking control. After frequency locking, the circuit of large-small driving force conversion is used to achieve fast step response. At the initial stage of step response, a large driving force is used to realize rapid increase of the amplitude of the signal output by the driving mode. After reaching the preset value, it turns to small driving force to achieve amplitude stability. The test results show that when the difference between the starting frequency and the resonant frequency is 2 Hz, the quick frequency locking time is reduced by 68% compared with the traditional method. Through the control method of fast step response, the step response time is less than 4 s. When the difference between the starting frequency and the resonant frequency is 2 Hz, the total startup time for the driving mode amplitude to reach stability is less than 9 s. The total start-up time is reduced by more than 80%. The quick start-up of high quality disk resonant MEMS gyroscope is realized.

## Data Availability

Not applicable.
